# Hasty generalizations and generics in medical research: A systematic review

**DOI:** 10.1371/journal.pone.0306749

**Published:** 2024-07-05

**Authors:** Uwe Peters, Henrik Røed Sherling, Benjamin Chin-Yee

**Affiliations:** 1 Department of Philosophy, Utrecht University, Utrecht, Netherlands; 2 Faculty of Philosophy, University of Cambridge, Cambridge, United Kingdom; 3 Department of History and Philosophy of Science, University of Cambridge, Cambridge, United Kingdom; 4 Department of Medicine, Division of Hematology, Schulich School of Medicine and Dentistry, Western University, London, Canada; University of Ferrara: Universita degli Studi di Ferrara, ITALY

## Abstract

It is unknown to what extent medical researchers generalize study findings beyond their samples when their sample size, sample diversity, or knowledge of conditions that support external validity do not warrant it. It is also unknown to what extent medical researchers describe their results with precise quantifications or unquantified generalizations, i.e., generics, that can obscure variations between individuals. We therefore systematically reviewed all prospective studies (*n* = 533) published in the top four highest ranking medical journals, *Lancet*, *New England Journal of Medicine* (*NEJM*), *Journal of the American Medical Association* (*JAMA*), and the *British Medical Journal* (*BMJ*), from January 2022 to May 2023. We additionally reviewed all *NEJM Journal Watch* clinical research summaries (*n* = 143) published during the same time. Of all research articles reporting prospective studies, 52.5% included generalizations beyond specific national study populations, with the numbers of articles with generics varying significantly between journals (*JAMA* = 12%; *Lancet* = 77%) (*p* < 0.001, *V* = 0.48). There was no evidence that articles containing broader generalizations or generics were correlated with larger or more nationally diverse samples. Moreover, only 10.2% of articles with generalizations beyond specific national populations reported external validity strengthening factors that could potentially support such extrapolations. There was no evidence that original research articles and *NEJM Journal Watch* summaries intended for practitioners differed in their use of broad generalizations, including generics. Finally, from the journal with the highest citation impact, articles containing broader conclusions were correlated with more citations. Since there was no evidence that studies with generalizations beyond specific national study populations or with generics were associated with larger, more nationally diverse samples, or with reports of population similarity that may permit extensions of conclusions, our findings suggest that the generalizations in many articles were insufficiently supported. Caution against overly broad generalizations in medical research is warranted.

## Introduction

Translating medical research to practice rests crucially on the external validity of results, i.e., on the generalizability of findings from study samples to target populations [1]. More generalizable findings apply to more people, making them more valuable for clinicians and policymakers in the real world [2,3].

To avoid misinterpretations about the generalizability of results, medical researchers need to tailor their conclusions to the evidence and the population(s) to which the results obtained with particular samples are meant to apply [4]. Testing more rather than fewer participants will make inferences more robust and more widely applicable as this can reduce random variation, increasing the probability that a statistically significant result reflects a true effect also likely to occur in retesting with similar groups [5]. Moreover, while homogenous samples can be preferable to better control for confounders in research aiming to identify causal mechanisms [6], after discovering a causal mechanism, researchers still need to identify conditions that modify its effects. This requires testing for differences in effects between people, calling for more diverse samples than those initially tested [3,7]. Alternatively, if researchers with small and homogenous samples wish to generalize about causal mechanisms beyond their sample, they would need to offer reasons for assuming that the mechanisms are stable across populations, or that their sample and target populations are relevantly similar [8].

Generalizations of results from a sample to a broader population when the sample is not sufficiently large or diverse, or when researchers have not offered support for thinking their sample and target population are similar enough to warrant the extrapolation are often called *hasty generalizations* [8–10]. To help reduce hasty generalizations in medical research, author guidelines often recommend a conservative approach. The American Medical Association (AMA), for example, states in its author guidelines that study “conclusions should be based on the study results and limited to the specific *population* represented by the study *sample* [emphasis in original]” [11, p. 1007].

However, even when their samples are large and representative, and conclusions are limited to specific categories of people, medical researchers may still generalize too broadly if they use statements that gloss over individual differences (e.g., in treatment efficacy). This is because in clinical trials, individual outcomes within tested groups are aggregated but outcomes between individuals are rarely the same (e.g., the variance in individuals can be up to four times greater than in groups [12,13]). Concluding statements that obscure individual variability are therefore inherently problematic and can be considered a subset of hasty generalizations.

One such type of statements are *generics*, which are present tense indicative mode generalizations with a subject-predicate phrase that refers to categories of individuals (e.g., “people with OCD”), treatments, or abstract phenomena (e.g., “mindfulness reduces anxiety”) without a quantifier (e.g., “some”, “75%”) [14,15]. Generics are problematic in science communication because they are semantically underdetermined (e.g., the generic “people with OCD benefit from CBT” may refer to some, many, or all people with OCD), implying broad, timeless conclusions while disguising variability, risking misinterpretation [15–17].

However, despite increasing attention to the quality of clinical trial reporting [19–21] and generics use in science [15,17,18], it remains unclear to what extent medical researchers produce hasty generalizations, how impactful articles with such generalizations are, and whether the generalizations in primary research articles differ in their scope (e.g., generics use) from those found in research summaries written for practicing clinicians.

Investigating these questions is critical. If medical researchers make claims that are broader than warranted by the evidence or that mask variations, and if articles with such claims are impactful and summarized for practitioners without adjustments in generalization scope, this may contribute to regulatory approval or clinical use of treatments that are ineffective or harmful for people [3,19].

We therefore conducted a systematic review of medical articles to investigate the presence of hasty generalizations, measuring them in two ways. First, we analyzed whether articles with broader result claims (i.e., statements describing a study’s findings, or making recommendations based on them) were associated with larger, more nationally diverse samples, or reports of generalizability strengthening factors. We treated the absence of such associations as indicative of hasty generalizations because broader result claims tend to require larger, more nationally diverse samples, or generalizability supporting background assumptions [8,12]. Second, we operationalized hasty generalizations as present tense result claims with a majority quantifier (e.g., “most”) or a generic about a nationally unspecified population when the researchers had only sampled people with a specific nationality and did not report factors that could support extrapolations beyond this population.

We aimed to answer five research questions (RQs):

*RQ1*. Do medical researchers limit their conclusions to the specific population represented by the study sample, and how common are generics in result claims of medical articles?
*RQ2*. Are articles with broader conclusions (e.g., generics) associated with larger, more nationally diverse samples than those without?
*RQ3*. Do medical researchers consider in their articles whether their samples and the population(s) to which results are generalized are, in relevant respects, similar to warrant the generalization?
*RQ4*. Are studies with broader conclusions associated with higher citation impact?
*RQ5*. Do the generalizations in primary research articles differ in their scope (e.g., use of generics) from those found in research summaries intended for practicing clinicians?

To further clarify the problematic generalizations in focus here, two subtly different ways in which authors may inappropriately characterize populations can be distinguished. (1) While their data may involve a specific sample (e.g., 100 US smokers), authors may generalize their conclusion to a whole category of people (e.g., “all smokers”, “smokers”, “US smokers”). (2) While the sample may be limited or unrepresentative (e.g., a study on smokers may include only US males), authors may characterize the sample in a way that invokes a broader category that does not mention the limitations (e.g., “smokers”, not “US male smokers”).

Cases of (1) include many cases of (2) (e.g., when authors only sampled 100 US smokers but then characterize the sample when concluding more broadly as “smokers”). But cases of (1) do not include all cases of (2): (2) could happen when one is simply describing the specific sample (e.g., in the Methods) and not generalizing results. Conversely, not all cases of (1) are also cases of (2): when authors only sampled 100 white male smokers and then generalize to “white male smokers”, this is a case of (1) but need not be a case of (2) because relevant limitations (being white and male) are mentioned.

Our study focuses primarily on cases of (1) but also includes cases of (2) where authors move from samples with a specific nationality to generalizations that do not mention nationality. We did not consider cases of (2) where authors move from samples with, for instance, specific gender or age to generalizations that do not mention gender or age.

## Methods

This systematic review is reported following the Preferred Reporting Items for Systematic Reviews and Meta-Analyses Statement (PRISMA) [20]. The study was preregistered on an Open Science Framework (OSF) platform. The preregistration, all materials, and data are available here. We adapted previously published research protocols [8,15].

*Search strategy*. To identify relevant studies, we selected all general medicine journals in the list of the ten overall highest impact journals according to the 2022 Journal Citation Reports, resulting in four outlets: *The Lancet*, *New England Journal of Medicine* (*NEJM*), *Journal of the American Medical Association* (*JAMA*), and *The British Medical Journal* (*BMJ*). To compare the generalizations in primary research articles with the generalizations in independent research summaries that were written about these articles, we used *NEJM Journal Watch*, a popular outlet among clinicians that specializes in publishing research summaries of clinical articles [21] (‘research summaries’ henceforth refers specifically to *NEJM Journal Watch* publications). We searched for studies and summaries published between January 1, 2022, and May 22, 2023.

*Study selection*. From the four medical journals, we included all prospective studies. Retrospective studies, meta-analyses, and systematic reviews were excluded. From *NEJM Journal Watch*, we included all summaries of prospective studies published in the four selected journals during our timeframe. Summaries of retrospective studies, meta-analyses, systematic reviews, and guidelines were excluded.

*Data extraction*. Three researchers did the data extraction and coding. For each article, we extracted title, journal name, article impact, operationalized as Google Scholar citation count [22], sample size (total analyzed sample), and sample diversity focusing only on sample country or region. For feasibility, we set aside other dimensions of sample diversity (e.g., gender, race, age). Based on sample country or region, we categorized samples as Western or non-Western using previously established geographical categorizations [23,24]. Samples with higher (versus lower) country or region counts were defined as more (versus less) nationally diverse.

Furthermore, we identified all result claims from each entire article (title, highlights, abstract, introduction, discussion, and conclusion), extending prior studies that analyzed only title, highlights, and abstracts [15]. (Result claims were claims reporting a study’s own original findings, not findings that authors reviewed, e.g., in their background sections.) We then determined each result claim’s *scope of conclusion*, i.e., the range of people to which the claim referred, classifying claims as either *restricted* or *generalized*. Claims defined as *restricted* did not extrapolate findings beyond the study sample or study population (i.e., the specific population that researchers take to be directly represented by the study sample), or used minority quantifiers (e.g., “some European patients”) or past tense to constrain their scope. Claims defined as *generalized* were not limited in any of these ways but belonged to one or more of the following three types of statements (for details, see OSF material):

1. *Unrestricted claims*. These were present tense indicative sentences such as (a) majority quantified statements about people (e.g., “most people with *X* benefit from *Y*”), (b) generics (e.g., “the study suggests that people with *X* benefit from *Y*” (‘framed generics’), or “people with *X* benefit from *Y*” (‘bare generics’ [15])), or (c) any other open scope statement that suggested the results applied beyond study participants either to broader populations described with a generic noun phrase (e.g., “our results validate *Y* as a therapy for patients with *X*”), or to no particular population (e.g., “there are benefits of treatment *Y*”).
2. *Hedged claims*. These were claims of type (a), (b), or (c) that contained modal verbs (e.g., “may”, “can”), or qualifiers such as “seems”, “has the potential”, or “is likely”.
3. *Practice-related claims*. These were treatment recommendations that presupposed a generalization of study results to most or all patients with a particular condition or people as such (e.g., “our results support doing *Y*”, “women with a tubal ectopic pregnancy should not be offered gefitinib”).

All generalized claims were extracted, copied to a spreadsheet (available here), and coded for whether they mentioned sample country or region (e.g., “US smokers”). This information allowed us to examine if result claims were broader than warranted by a sample’s national diversity (e.g., when the sample was only US smokers but result claims spoke of “smokers”, “all smokers”).

If an article contained only restricted claims, the article itself was coded as *restricted*. If the article contained at least one generalized claim, the article itself was coded as *generalized*.

Generics were a subset of unrestricted and hedged generalized claims. We coded articles on whether and how often they contained result claims with generics, adapting previously used generics classification criteria [8,15]. We opted for the more comprehensive concept of generalized claims as our main category (rather than generics) because hasty generalizations may also become expressed in unrestricted statements without generics, or in recommendations.

Additionally, we coded whether articles reported external validity limitations, and external validity strengthening factors, including background assumptions, previous findings, or study or sample features that may permit extrapolations from study samples to broader, specifically, nationally different populations (e.g., representative samples, genetic, clinical, etc. similarity between populations). Finally, we extracted the scope of conclusions in *NEJM Journal Watch* research summaries using the same coding method.

### Data analysis

After the data coding, inter-rater agreement between the three coders was calculated using Cohen’s κ, which, across variables, was consistently above substantial (κ = 0.80, 95% CI 0.75 to 0.85, to κ = 0.97, 95% CI 0.94 to 0.99). For the scope of conclusion variable (including the generics coding), we additionally asked two project-naïve coders to apply our pre-specified criteria to 25% of the data. Inter-rater agreement between their and our classifications was high (κ = 0.88, 95% CI 0.80 to 0.95, to κ = 0.89, 95% CI 0.81 to 0.96). Remaining disagreements were resolved by discussion.

For the analyses, we focused on both the number of restricted and generalized articles per journal, and the number of generalized claims (including generics) within articles. To examine associations between generalized (versus restricted) articles and sample size and national diversity, we used an article’s scope of conclusion (restricted/generalized) as a categorical independent variable and sample size, and country or region count as numerical dependent variables.

Non-parametric statistics (χ^2^, Kruskal-Wallis *H*, Mann-Whitney *U*, rank-biserial correlation, Spearman correlation, and ϕ coefficient tests) were used. The data violated the normality assumption and non-parametric statistics are more conservative yielding more robust findings. For the impact analysis, we followed previous studies, identified each article’s online publication date, and normalized the article’s total citation count by calculating the Relative Citation Rate (RCR) [25], before using the RCR as a continuous dependent variable. We set α to 0.05. Tests were two-tailed and performed with JASP and IBM SPSS 29.0.

## Results

**Fig 1:**
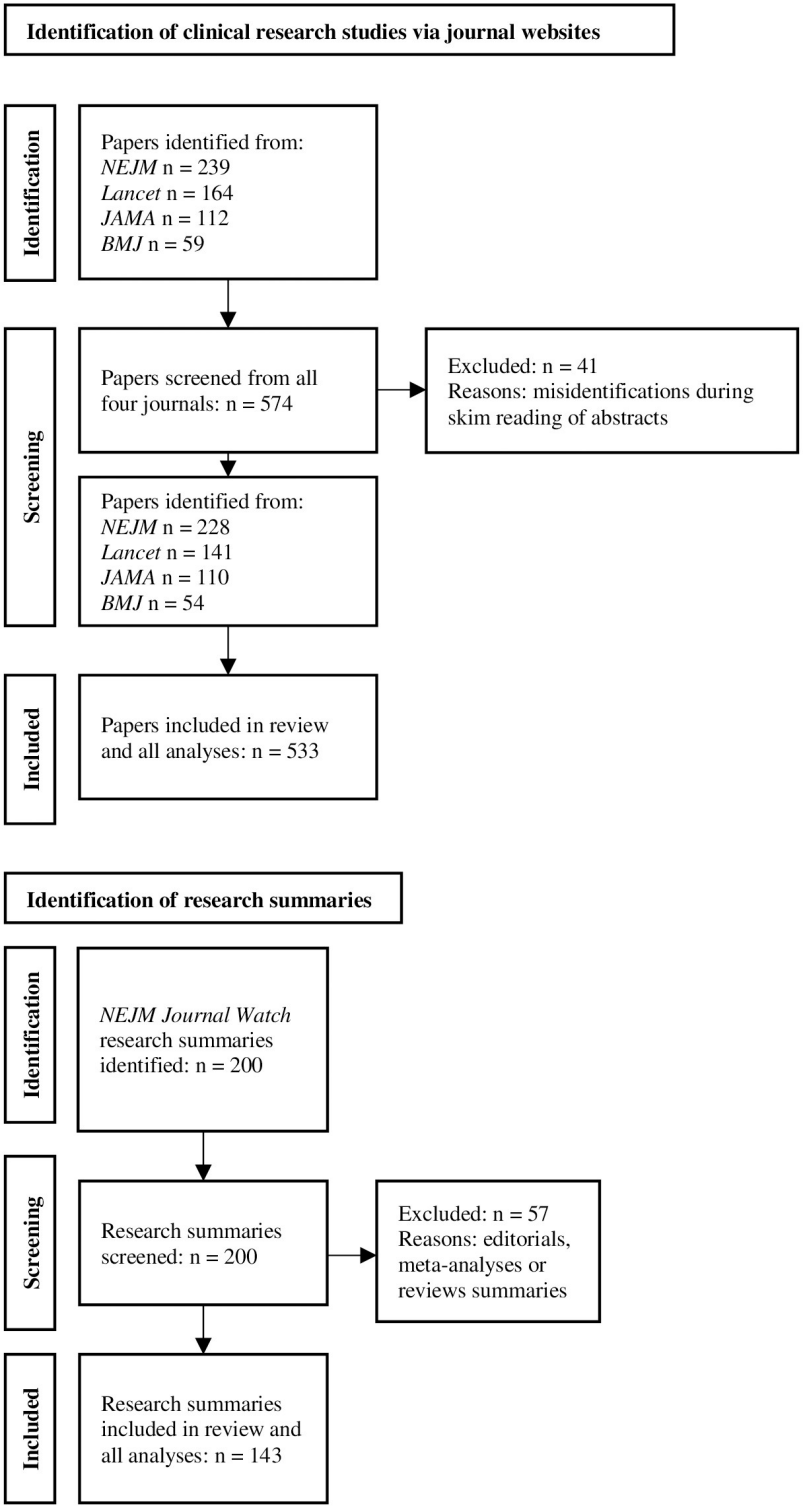
PRISMA flowcharts of the systematic reviews.

Our initial search identified 574 articles. 533 met inclusion criteria for full-text analysis. Among the *NEJM Journal Watch* research summaries, the initial search yielded 200 results. 143 (published across 35 *NEJM Journal Watch* issues) met inclusion criteria (see Fig 1).

Descriptive details of the 533 clinical research studies are shown in Table 1 (and in S1–S4 Tables).

**Table 1:** Article-level descriptive details per journal.

Articles by journal (*n*, %)	*Lancet*	*NEJM*	*JAMA*	*BMJ*
Total *N*	141	228	110	54
Median # of participants (IQR)	810.0 (291.0–1778.0)	559.5 (178.5–1673.5)	694.5 (323.0–1373.3)	1482.0 (527.0–23639.0)
Median # of countries/regions	2.0 (1.0–11.0)	3.0 (1.0–9.8)	1.0 (1.0–2.0)	1.0 (1.0–1.0)
Sample composition				
Non-Western	26 (18.4)	26 (11.4)	14 (12.7)	6 (11.1)
Mixed	51 (36.2)	79 (34.6)	13 (11.8)	3 (5.6)
Western	64 (45.4)	123 (53.9)	83 (75.5)	45 (83.3)
External validity				
Limitations reported	78 (55.3)	144 (63.2)	76 (69.1)	37 (68.5)
Strengthening factors reported	36 (25.5)	47 (20.6)	20 (18.2)	20 (37.0)
Publication year				
2021	2 (1.4)	0	0	0
2022	94 (66.7)	158 (69.3)	79 (71.8)	43 (79.6)
2023	45 (31.9)	70 (30.7)	31 (28.2)	11 (20.4)
Trial type				
Phase I	4 (2.8)	5 (2.2)	0	0
Phase II	18 (12.8)	44 (19.3)	2 (1.8)	0
Phase III	73 (51.8)	109 (47.8)	39 (35.5)	14 (25.9)
Phase IV	5 (3.5)	8 (3.5)	10 (9.1)	3 (5.6)
No details	41 (29.1)	62 (27.2)	59 (53.6)	37 (68.5)
Top three clinical subspecialties				
Infectious disease	27 (19.1)	44 (19.3)	16 (14.5)	8 (14.8)
Oncology	22 (15.6)	41 (18.0)	7 (6.4)	2 (3.7)
Cardiology	15 (10.6)	26 (11.4)	16 (14.5)	2 (3.7)

The results for each of our five research questions were as follows.

*RQ1*. *Do medical researchers limit their conclusions to the specific population represented by the study sample*, *and how common are generics in result claims of medical articles*? Of all 533 articles, 285 (53.5%) were generalized, containing at least one of the three kinds of generalized claims. 246 articles contained generics, representing 86.3% of all articles with generalized claims and 46.2% of all 533 articles. Moreover, only 5 of all the 285 generalized articles mentioned sample country or region in their generalized claims, meaning that 52.5% (*n* = 280) of all articles contained result claims or recommendations that were not limited to a specific national sample or study population. Table 2 presents three examples for each type of generalized claim.

**Table 2:** Examples of generalized claims from the sample. Numbers in brackets refer to the article number in the OSF data spreadsheet.

*Unrestricted claims*
1. “As a nationwide trial in the US, it is generalizable for all adults aged 30 years or older with COVID-19.” (120)
2.“EV71vac is safe, well-tolerated, and highly effective in preventing EV71 associated diseases in children aged 2–71 months.” (251)
3. “These findings suggest that highly processed foods are associated with poor health outcomes independently of their low nutritional composition, but not the other way around.” (31)
*Hedged claims*
1. “Gene therapy for hemophilia A may enable maintenance of steady, endogenous factor VIII activity without regular prophylaxis.” (380)
2. “Our data show that treatment with nirmatrelvir plus ritonavir early in Covid-19 illness can decrease progression to severe disease and quickly reduce SARS-CoV-2 viral load.” (411)
3. “The results suggest that artificial sweeteners might represent a modifiable risk factor for cardiovascular disease prevention.” (33)
*Practice-related claims*
1. “These data provide further evidence to support the use of an SGLT2 inhibitor as essential therapy in patients with heart failure, regardless of the presence or absence of type 2 diabetes mellitus or left ventricular ejection fraction.” (478)
2. “The results of our trial support the use of darolutamide in combination with androgen-deprivation therapy and docetaxel in patients with metastatic, hormone-sensitive prostate cancer.” (415)
3. “In light of our clinical trial results, we can confidently conclude that women with a tubal ectopic pregnancy should not be offered the combination of gefitinib and methotrexate because it is no more effective than treatment with methotrexate alone.” (322)

**Fig 2:**
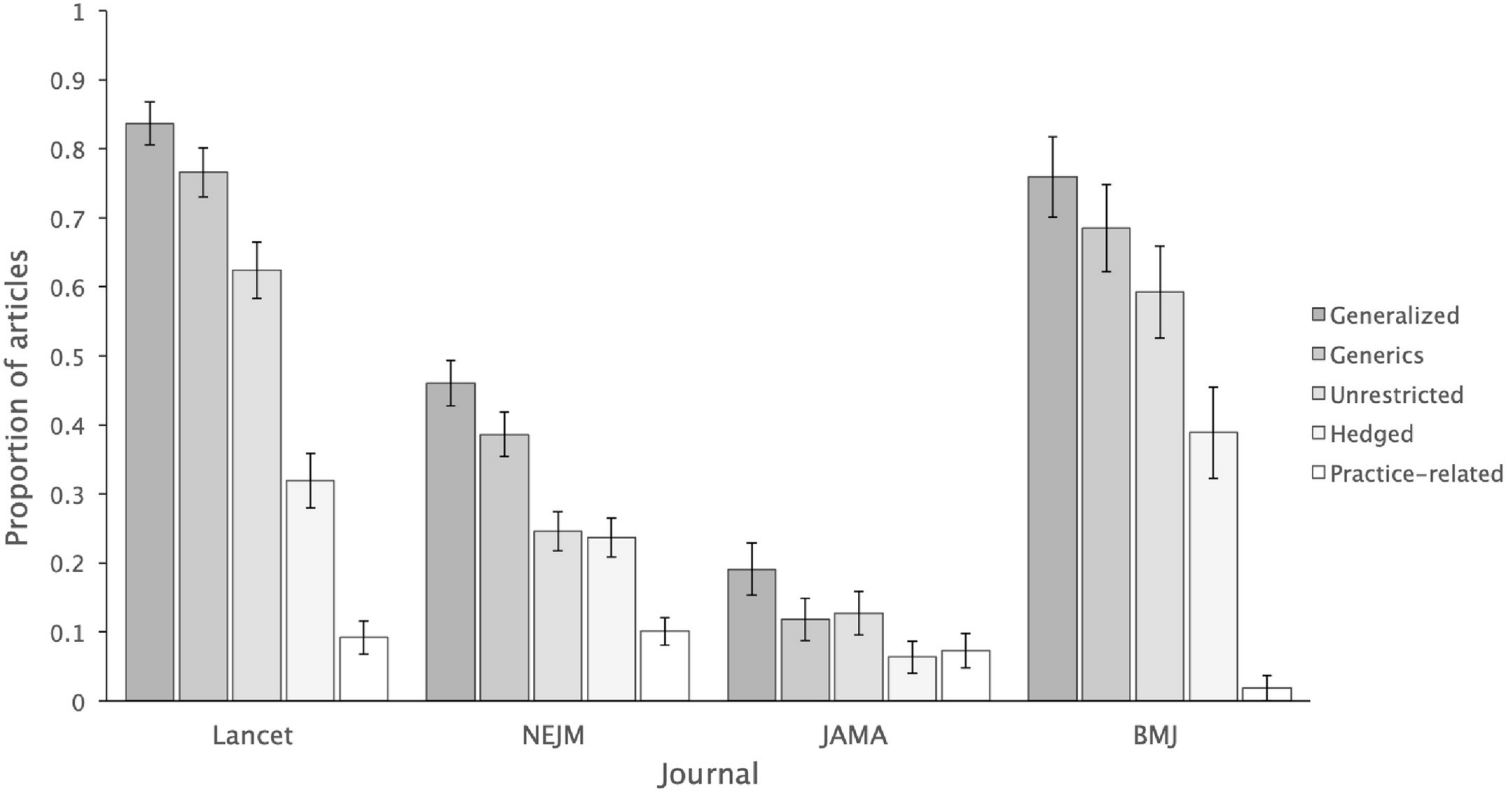
The proportion of articles with generalized, generic, unrestricted, hedged, or practice-related result claims (derived by dividing the number of articles with these claims with the total number of articles of each journal). Error bars indicate standard error for the variability in proportion estimates.

There were significant differences in the distribution of generalized and restricted articles between the four journals (χ^2^ (3) = 119.99, *p* < 0.001, *V* = 0.47), with *Lancet* having the highest proportion of generalized articles (83.7%) and *JAMA* having the lowest (19.1%) (see Fig 2).

Pairwise journal comparisons (Table 3) showed that *Lancet* had significantly higher counts of generalized versus restricted articles than *NEJM*, with the odds of having a generalized scope of conclusion being about 6 times higher for *Lancet* articles compared to those published in *NEJM* (OR = 6.01, 95% CI 3.58 to 10.08). This difference became more pronounced when *Lancet* was compared with *JAMA* (OR = 21.74, 95% CI 11.32 to 41.75). However, there was no evidence of such a difference between articles by *Lancet* and *BMJ*. Relatedly, *BMJ*, too, had higher generalized article counts than both *NEJM* (OR = 3.70, 95% CI 1.88 to 7.26) and *JAMA* (OR = 13.37, 95% CI 6.10 to 29.29), while *NEJM* had higher counts than *JAMA* (OR = 3.62, 95% CI 2.10 to 6.22) (Table 3).

**Table 3:** χ^2^ test results comparing journals in terms of their number of generalized articles and articles with generics. Bonferroni correction for multiple comparions α = 0.008.

Journal comparison	Generalized articles	χ^2^ (1)	*p*	ϕ
*Lancet* vs. *NEJM*	118 vs. 105	51.61	< 0.001	0.37
*Lancet* vs. *JAMA*	118 vs. 21	104.35	< 0.001	0.65
*Lancet* vs. *BMJ*	118 vs. 41	1.56	0.21	– 0.09
*NEJM* vs. *JAMA*	105 vs. 21	23.07	< 0.001	0.26
*NEJM* vs. *BMJ*	105 vs. 41	15.61	< 0.001	0.24
*JAMA* vs. *BMJ*	21 vs. 41	49.76	< 0.001	0.55
	**Articles with generics**			
*Lancet* vs. *NEJM*	108 vs. 88	50.52	< 0.001	0.37
*Lancet* vs. *JAMA*	108 vs. 13	103.85	< 0.001	0.64
*Lancet* vs. *BMJ*	108 vs. 37	1.34	0.25	– 0.08
*NEJM* vs. *JAMA*	88 vs. 13	25.39	< 0.001	0.27
*NEJM* vs. *BMJ*	88 vs. 37	15.84	< 0.001	0.24
*JAMA* vs. *BMJ*	13 vs. 37	54.94	< 0.001	0.58

The four journals also differed significantly in the distribution of texts with versus without generics (χ^2^ (3) = 120.87, *p* < 0.001, *V* = 0.48), with *Lancet* having the highest proportion of text with generics, 76.6%, and *JAMA* having the lowest, 11.8% (Fig 2). Pairwise comparisons (Table 3) showed that *Lancet* had significantly higher counts of articles with versus without generics than *NEJM* (OR = 5.21, 95% CI 3.25 to 8.35) and *JAMA* (OR = 24.42, 95% CI 12.15 to 49.07). However, no significant difference between *Lancet* and *BMJ* was observed. Furthermore, *NEJM* had higher counts of articles with generics than *JAMA* (OR = 4.69, 95% CI 2.48 to 8.87), while *BMJ* had higher counts than both *JAMA* (OR = 16.24, 95% CI 7.18 to 36.70) and *NEJM* (OR = 3.46, 95% CI 1.83 to 6.52).

**Fig 3:**
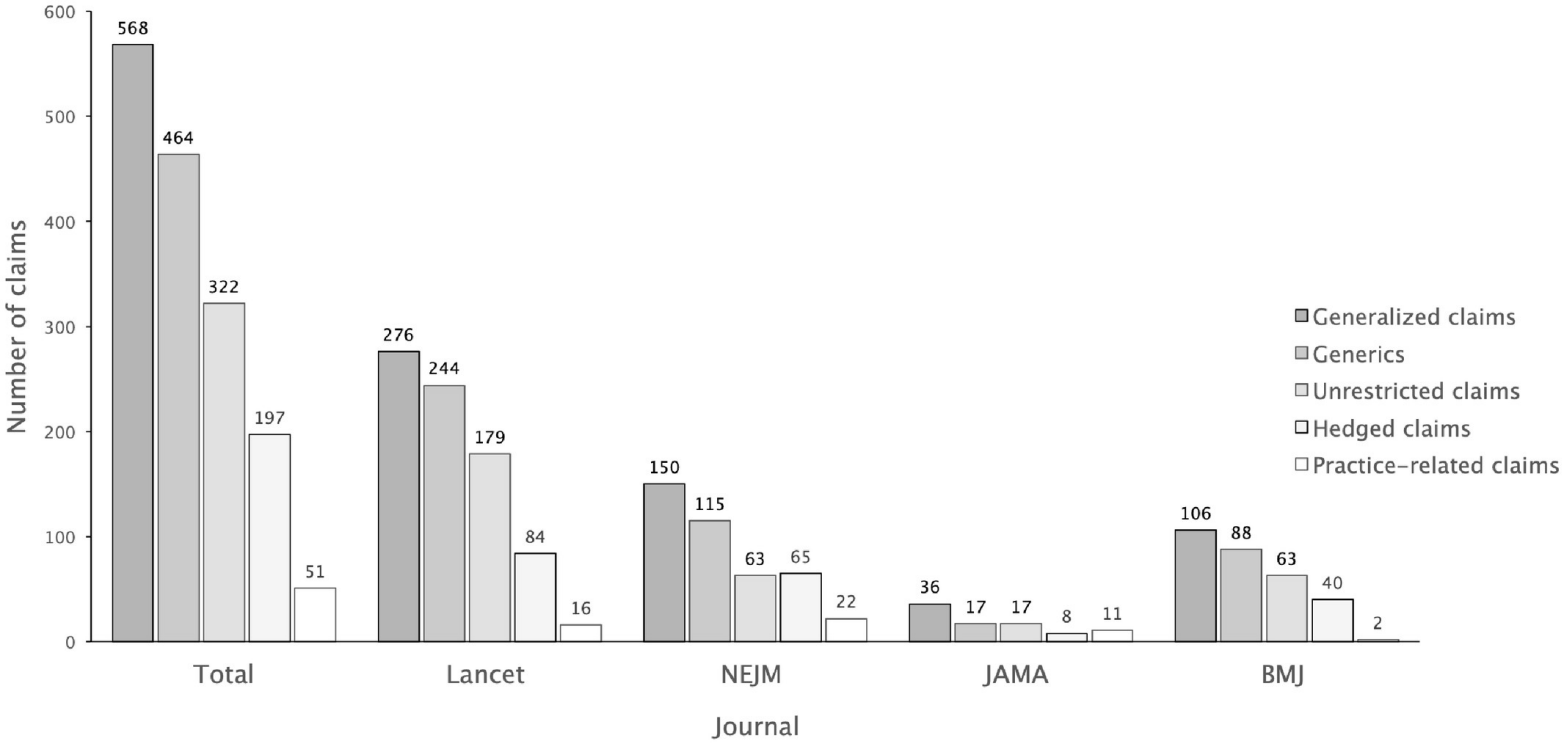
Number of generalized claims in total shown by claim type and journal.

Turning from article counts to the number of generalized claims, in all 285 generalized articles, we found a total of 568 generalized claims. The strongest type, unrestricted claims, were found in 190 (66.7%) of these articles, constituting 35.6% of all articles. Focusing specifically on the use of generics, as shown in Fig 3, 81.7% (*n* = 464) of all generalized claims were generics.

**Fig 4:**
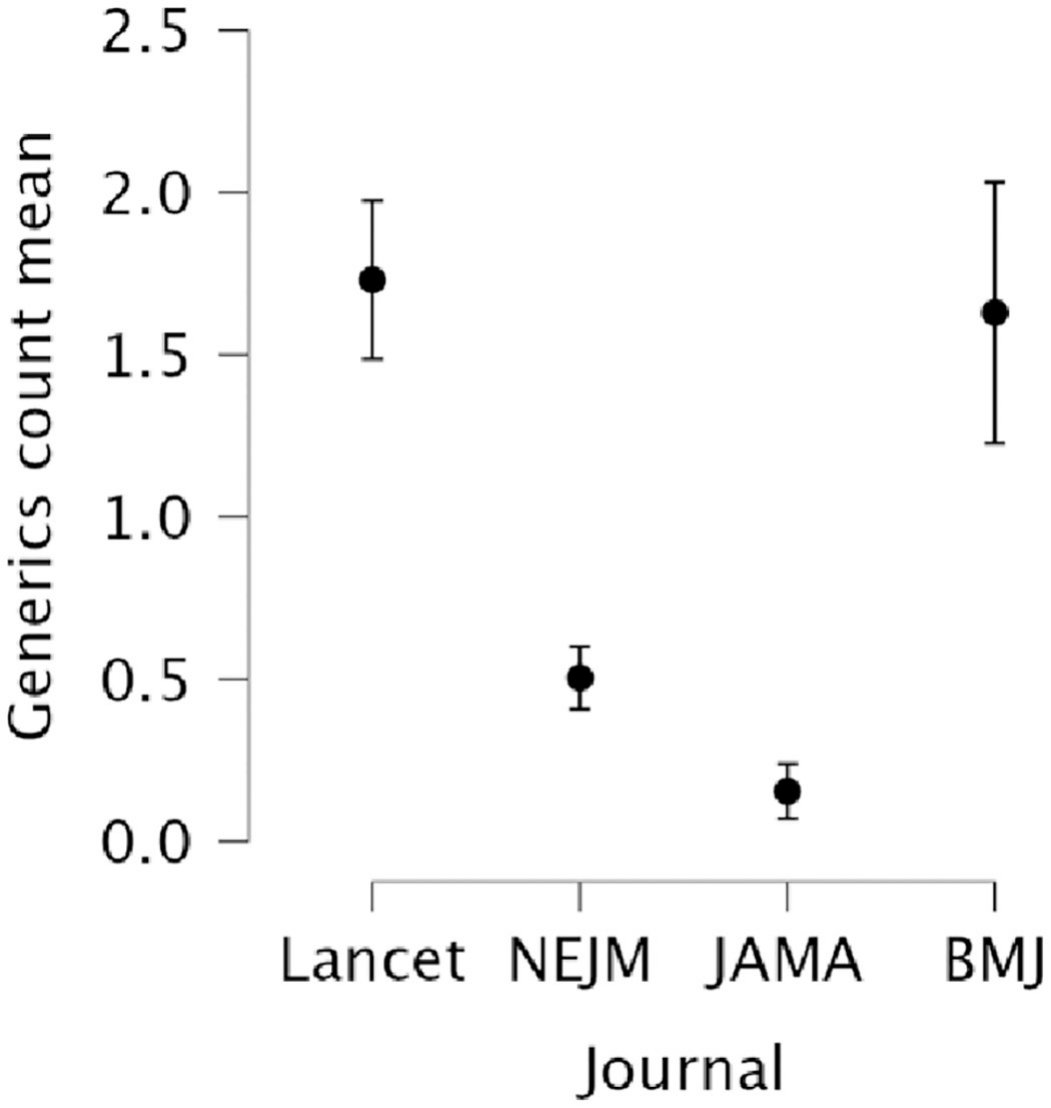
Interval plot showing the mean overall generics count of each journal’s total number of articles.

The counts of generics in result claims differed significantly between journals, with *Lancet* (*Mean rank* = 364.52) having the highest number followed by *BMJ* (*Mean rank* = 348.31), *NEJM* (*Mean rank* = 233.38) and *JAMA* (*Mean rank* = 171.77) (*H* (3) = 150.21, *p* < 0.001). Post hoc comparison results are shown in Table 4. Fig 4 presents the mean differences in generic counts of the articles by journal.

**Table 4:** Comparisons of the number of generics in articles by journal. Bonferroni correction α = 0.008.

Journal comparison	*Mean ranks*	*U*	*z*	*p*	*r*
*Lancet* vs. *NEJM*	243.54 vs. 148.80	7819.50	− 8.89	< 0.001	0.51
*Lancet* vs. *JAMA*	163.81 vs. 77.53	2423.50	− 10.13	< 0.001	0.69
*Lancet* vs. *BMJ*	99.17 vs. 94.95	3642.50	− 0.48	0.63	0.04
*NEJM* vs. *JAMA*	184.10 vs. 139.24	184.10	− 4.93	< 0.001	0.27
*NEJM* vs. *BMJ*	129.48 vs. 192.24	3416.00	– 5.66	< 0.001	− 0.45
*JAMA* vs. *BMJ*	66.00 vs. 116.12	1154.50	– 7.81	< 0.001	− 0.61

*RQ2*. *Are articles with broader conclusions associated with larger*, *more nationally diverse samples than those without*? Rank-biserial correlation tests did not find evidence that generalized articles (*r* =—0.09, *p* = 0.08) or articles with generics (*r* =—0.06, *p* = 0.22) were associated with larger samples, or with higher country or region counts (generalized articles: *r* =—0.05, *p* = 0.29; articles with generics: *r* =—0.03, *p* = 0.52). Results by journal are shown in Table 5.

**Table 5:** Association test results per journal using rank-biserial correlation and ϕ coefficient tests.

*Lancet* articles (*n* = 141)	*r*	ϕ	*p*
Scope of conclusion and sample size	0.04		0.76
Scope of conclusion and national sample diversity	− 0.04		0.74
Scope of conclusion and report of external limitations		0.03	0.74
Scope of conclusion and report of external validity strengthening factors		− 0.01	0.95
*NEJM* articles (*n* = 228)			
Scope of conclusion and sample size	− 0.06		0.42
Scope of conclusion and national sample diversity	− 0.05		0.48
Scope of conclusion and report of external limitations		0.01	0.85
Scope of conclusion and report of external validity strengthening factors		0.14	0.04
*JAMA* articles (*n* = 110)			
Scope of conclusion and sample size	− 0.07		0.61
Scope of conclusion and national sample diversity	0.10		0.39
Scope of conclusion and report of external limitations		− 0.08	0.43
Scope of conclusion and report of external validity strengthening factors		− 0.11	0.25
*BMJ* articles (*n* = 54)			
Scope of conclusion and sample size	− 0.13		0.48
Scope of conclusion and national sample diversity	0.16		0.23
Scope of conclusion and report of external limitations		0.09	0.53
Scope of conclusion and report of external validity strengthening factors		0.25	0.06

Regarding sample diversity, the overall three most sampled countries were the USA (273, 51.2%), UK (176, 33.0%), and Canada (129, 24.2%) (see also S2 Table). 201 (70.5%) of all 285 articles with generalized claims had only tested either Western (*n* = 154, 54.0%) or non-Western (*n* = 47, 16.5%) samples alone (S3 Table, S1 Fig). However, only in 5 of these 201 articles (one with non-Western sample; four with Western samples) did the generalized claims mention nationality. Therefore, in 194 articles (68.1% of all 285 generalized articles) researchers had either only a Western or non-Western sample but did not limit their conclusions to it.

*RQ3*. *Do medical researchers consider in their articles whether their samples and the population(s) to which results are generalized are*, *in relevant respects*, *similar to warrant the generalization*? In 335 (62.9%) articles, researchers mentioned limitations to the generalizability of their results. However, in 175 (52.2%) of them, they still produced generalized claims, most of which contained generics (*n* = 149, 85.1%). There was no evidence that articles with reports of external validity limitations were associated with being restricted rather than generalized articles (ϕ = –0.03, *p* = 0.46), or with not containing rather than containing generics (ϕ =—0.04, *p* = 0.31).

However, generalized articles (*n* = 285) compared to restricted articles were associated with reporting external validity strengthening factors that may support generalizations from sample to study population or beyond (ϕ = 0.11, *p* = 0.01, OR = 1.70, 95% CI 1.12 to 2.57). But there was no evidence that the same held for articles with generics (ϕ = 0.09, *p* = 0.04; Bonferroni correction α = 0.025). Results by journal are presented in Table 5.

Moreover, of all generalized articles, only 78 (27.4%) reported external validity strengthening factors, and only in 29, i.e., 10.2% of these articles did the relevant factors concern generalizations across nationalities potentially supporting conclusions beyond the specific national sample. Since, as noted, in only 5 generalized articles, the generalized result claims mentioned nationality, this means that at best only 34 generalized articles contained a reference to a specific national population in the result claims, or evidence that researchers considered whether their samples and the population(s) to which results were generalized were relevantly similar to warrant the generalization. Hence, in most, i.e., 251 (88.1%) of the generalized articles, which constitute 47.1% of all 533 articles, researchers did not offer reasons to believe that their samples were relevantly nationally similar to the larger populations to which they generalized.

*RQ4*. *Are studies with broader conclusions associated with higher citation impact*? Overall, generalized articles (*Mean rank* = 279.27) were associated with higher impact (normalized citation count) than restricted articles (*Mean rank* = 252.90), although the difference was only marginally significant (*U* = 31843.00, *z* =—1.97, *p* = 0.049, *r* =—0.10). There was no evidence of such an association between articles with generics (*Mean rank* = 269.24) versus without them (*Mean rank* = 265.08) and higher impact (*U* = 34769.00, *z* =—0.311, *p* = 0.75, *r* =—0.02).

Journal comparisons with Bonferroni correction (α = 0.01) indicated that for *NEJM*, which had the articles with the highest overall impact (see also S5 Table, S2 Fig), generalized articles (*Mean rank* = 128.25) had significantly higher impact than restricted articles (*Mean rank* = 103.13) (*U* = 5058.50, *z*– 2.82, *p* = 0.005, *r* =—0.22). There was no evidence that this was also the case for *Lancet* (*Mean rank* = 71.20 vs. *Mean rank* = 69.98, *U* = 1333.50, *z* =—0.13, *p* = 0.90, *r* =—0.02), *JAMA* (*Mean rank* = 58.00 vs. *Mean rank* = 54.91, *U* = 882.00, *z* =—0.40, *p* = 0.69, *r* =—0.06), or *BMJ* articles (*Mean rank* = 27.98 vs. *Mean rank* = 26.00, U = 247.00, z =—0.40, *p* = 0.70, *r* =—0.07).

Additionally, for *NEJM* articles, the more generalized claims an article contained, the higher its citation impact (*r*_s_ = 0.18, *p* = 0.005). But there was no evidence of such a correlation for *Lancet* (*r*_s_ =—0.07, *p* = 0.43), *JAMA* (*r*_s_ = 0.04, *p* = 0.71), *BMJ* (*r*_s_ = 0.14, *p* = 0.30), or all 533 articles combined (*r*_s_ = 0.04, *p* = 0.38). Similarly, focusing on the number of generics in generalized claims, there was no evidence that higher generics counts were associated with higher impact for all articles combined (*r*_s_ = 0.01, *p* = 0.79), or individually for *NEJM* (*r*_s_ = 0.11, *p* = 0.11), *Lancet* (*r*_s_ =—0.14, *p* = 0.10), *JAMA* (*r*_s_ = 0.04, *p =* 0.72), or *BMJ* (*r*_s_ = 0.07, *p* = 0.62).

**Fig 5:**
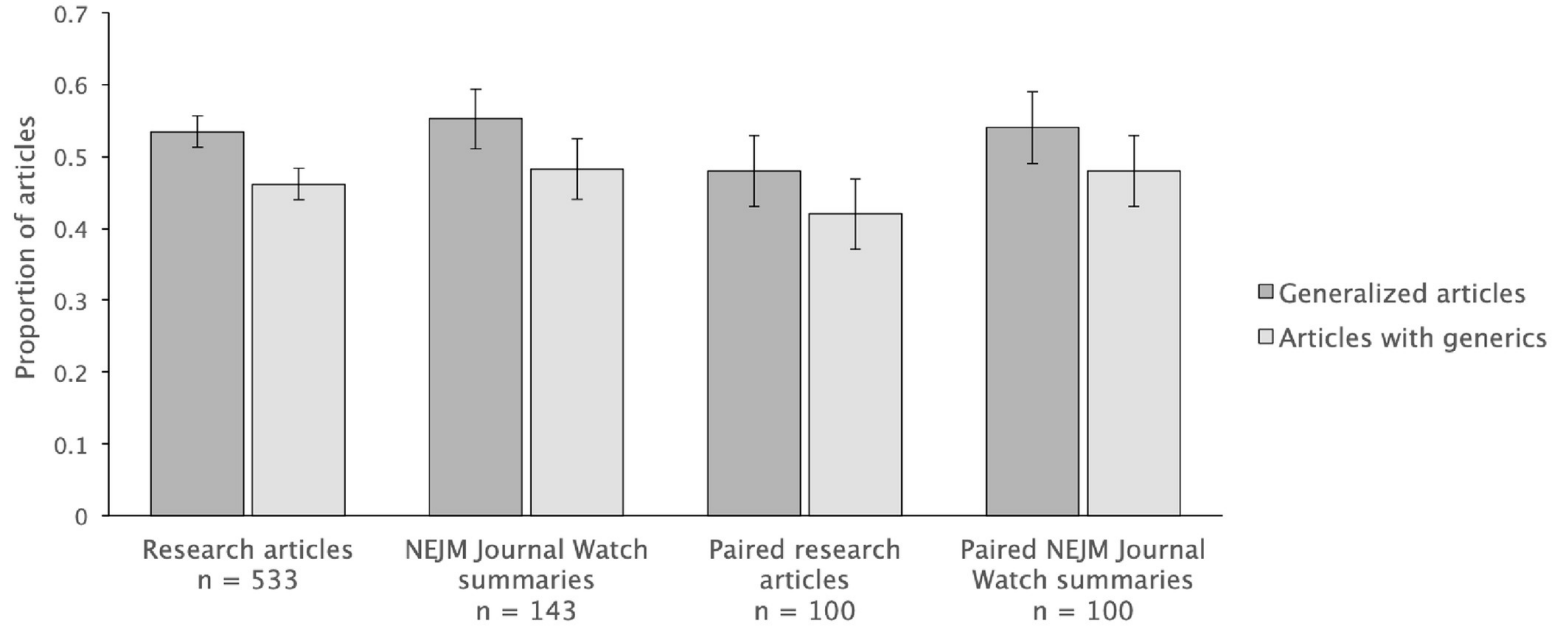
Comparisons of the proportions of clinical research articles and *NEJM Journal Watch* research summaries with generalized claims. Error bars indicate standard error for the variability in proportion estimates.

*RQ5*. *Do the generalizations in primary research articles differ in their scope from those found in research summaries intended for practicing clinicians*? Of 143 *NEJM Journal Watch* research summaries, 79 (55.2%) contained generalized claim and 69 (48.3%) contained generics. No evidence was found that research summaries differed significantly from primary research articles in the numbers of generalized or restricted texts (χ^2^ (1) = 0.14, *p* = 0.71, ϕ = 0.02) or in the number of texts with generics (χ^2^ (1) = 0.20, *p* = 0.66, ϕ = 0.02). Fig 5 presents the proportions by text type. There was also no evidence that research articles (*Mean rank* = 341.16) or summaries (*Mean rank* = 328.58) had overall statistically more generalized claims (*U* = 36691.00, *z* =—0.730, *p* = 0.47), or generics (*Mean rank* = 339.96 vs. *Mean rank* = 333.10, *U* = 37338.00, *z* =—0.41 *p* = 0.68) in the texts.

Among the 143 *NEJM Journal Watch* research summaries, 100 summaries were about primary research articles in our data set. When they were directly paired with each other, there was still no evidence of a significant difference between the two types of publications in overall counts of texts containing generalized claims (χ^2^ (1) = 0.72, *p* = 0.40, ϕ = 0.06) or generics (χ^2^ (1) = 0.73, *p* = 0.39, ϕ = 0.06) (Fig 5). Finally, there was also no evidence that the summaries (*Mean rank* = 101.47) differed significantly from their corresponding primary research articles (*Mean rank* = 99.54) in the number of generalized claims (*U* = 4903.50, *z* =—0.26, *p* = 0.80) or generics in articles (*Mean rank* = 101.20 vs. *Mean rank* = 99.80) (*U* = 4930.00, *z* =—0.19, *p* = 0.85).

## Discussion

Our analysis of clinical studies and research summaries reveals four key findings related to hasty generalizations, i.e., conclusions about broad populations when the sample is not sufficiently large or diverse, or when the researchers have not offered support for assuming the sample and target population are similar enough for the extrapolation.

First, in most articles (52.5%), medical researchers made claims or recommendations that generalized results beyond the specific national population sampled to all patients with a particular condition, or people in general. Result claims that go beyond the specific population sampled conflict with the AMA reporting guidelines that state that “conclusions should be based on the study results and limited to the specific *population* represented by the study *sample*” [11, p. 1007].

Second, we found no evidence that generalized articles were correlated with larger, more nationally diverse samples. In fact, in about 71% of all generalized articles, authors had sampled only Western or only non-Western populations. Researchers with small, homogenous samples might still be justified in generalizing to more diverse populations if they offer reasons for assuming relevant similarity between populations [8]. However, in over 88% of the generalized articles, authors did not mention external validity strengthening factors concerning generalizations across nationalities. Our findings therefore suggest that in these articles, which form about 47% of all 533 articles, the broad scope of result claims was insufficiently supported, indicating that hasty generalizations in medical research may have been common [10].

This can have harmful consequences. If researchers conclude in their articles that a treatment is safe, but they only sampled a Western population and did not consider whether being Western may influence relevant causal mechanisms, clinicians reading the article may come to use unsafe treatments for non-Western individuals, as ethnicity and Western background have been found to affect many clinical outcome measures [3]. For instance, studies found that clopidogrel was less effective in Pacific Islanders [26], 5-fluorouracil caused toxicities more often in Black than in White individuals [27], and the toxicity of the anticancer drug S-1 differed in European and US patients [28]. Due to genetic differences in pharmacokinetics and pharmacodynamics, clopidogrel [29], phenytoin [30], warfarin [31], clozapine [32], and carbamazepine [33] also had variable efficacy and side-effect profiles in Western and non-Western individuals. These findings highlight that relevant similarities between the populations cannot always be taken as given but may need to be supported through the testing of diverse groups [3] or an explicit articulation of background assumptions that warrant generalizations across populations.

Third, even if we had encountered correlations between broader conclusions and larger, more nationally representative samples, or if all conclusions had contained details about population nationality, our results would still indicate potentially problematic generalizations in clinical articles, because in about 46% of all articles, researchers reported findings by using generics. The use of generics in medical articles can be risky because, unlike quantified generalizations about a population, generics obscure differences between individuals within clinical groups and underdetermine prevalence, making claims inherently harder to test [8,15,17]. Generics also convey varying levels of a property’s prevalence: While “ravens are black” means all ravens are black, “mosquitos carry malaria” is true even though less than 10% of all mosquitos carry malaria, indicating that generics can allow for many exceptions [34]. These features of generics can cause miscommunication, as clinicians may need more background information to determine what prevalence level any given generic conveys [17].

Our finding of frequent generics use in scientific articles aligns with previous corpus analyses that found generics in 70%–89% of studies in, for instance, psychology and experimental philosophy [8,15,18]. However, since the overall frequency of articles with generics in our corpus of medical articles was below 50%, generics use in result claims may be much lower in top medical articles than in research articles in other fields—notably, only about 12% of *JAMA* articles contained generics in the result claims. This difference may be an underestimate because some of the previous studies [e.g., 15] coded only titles, research highlights, and abstracts, whereas we analyzed full articles, further suggesting systematic differences in generics use between some fields.

As noted by DeJesus et al., editorial policies can impact generalizations in psychology journals, with structural requirements such as the inclusion of short research highlights or the absence of formal demographic reporting policies potentially promoting generics use [15,18]. Correspondingly, variation in editorial guidelines between journals may help explain the overall lower generics use we observed in our study. For example, *JAMA*, which had the lowest percentage of articles with generics and overall generics count, explicitly instructs authors to “provide only conclusions of the study that are directly supported by the results”, while “avoiding speculation and overgeneralization” [35]. By contrast, *Lancet*, which had the highest percentage of articles with generics (about 77%, Fig 2), may inadvertently promote broader generalizations by encouraging authors to “provide a *general interpretation* of the results [emphasis added] and their significance rather than reiterating them”, without specific editorial injunction against overgeneralizations [36].

Other journal specific constraints on article formatting, including differences in the required length of titles, abstracts, individual sections, or whole articles, and even different cultural background, age, or gender (e.g., research found that women were more cautious about the importance of their findings [37]) by authors, reviewers, editors may also contribute to the variation in generalization types in articles between journals. Further research is required to establish the impact of these factors on generalizations in medical research and across disciplines.

Relatedly, our finding that researchers still produced generalized claims (85% containing generics) in more than 52% of articles despite having reported limitations to the generalizability of results calls for an explanation. Work in cognitive science suggests that scientists might sometimes be subject to a “generalization bias”, a tendency to unintentionally generalize findings even in the absence of sufficient evidence [38]. Our finding aligns with and may potentially be explained by the operation of such a bias among medical researchers.

Finally, it is often assumed that broader, exaggerated claims may attract more attention [15,39]. One might thus predict that articles with broader conclusions have higher citation impact. However, in our study, overall, the links between articles with generalized claims or generics and higher citation rates were either only marginally significant or not significant. Only the generalized articles published by *NEJM* were associated with higher impact. No association between articles with generics or generics counts and higher citations were observed. Our results are consistent with previous research that found exaggerated conclusions in up to 40% of medical press releases but did not find evidence that they were linked with an increased likelihood of news coverage, another measure of impact [39]. Notably, when generalized claims are well supported such claims may be reliable indicators of scientific success. But, if, as a result, their use in science is incentivized, this can have the unintended consequence of promoting overgeneralizations [38].

## Strengths, limitations, and recommendations

Our study has several strengths. While previous work on problematically broad generalizations in scientific articles focused on generics [15], our study also investigated the distribution of quantified or practice-related statements that may previously have been overlooked but may be particularly relevant and consequential in medical articles, which are often consulted by health care professionals for recommendations on treatments [17]. Moreover, unlike previous studies [8,15], our study compared the distribution of generics and other problematic generalizations in primary research article with their distribution in secondary research summaries thus extending the analysis to a new text format. Additionally, extending previous work [15], we coded whole articles (e.g., not just titles, research highlights, or abstracts) for generics to better capture their overall distribution. Also, we have documented all generalized claims in a spreadsheet here for other researchers to reproduce our findings or build on the dataset for their own studies.

Turning to limitations, when examining whether authors based their generalizations on background assumptions about population similarity, or other generalizability strengthening factors, we focused only on whether these factors were reported in articles. Authors might have had relevant background knowledge supporting their generalizations without making it explicit. However, if key generalizability strengthening (or limiting) factors remain implicit in articles, since not all readers may share the relevant tacit common ground in specific fields, the generalizations in the articles might still be insufficiently specified to avoid misinterpretation, suggesting the scope of the generalizations remains problematic [17].

Another limitation is that we did not code for demographic features other than nationality, for instance, gender or age. We therefore could not examine whether authors also overlooked gender or age in their generalizations. It might be that many generalized claims did mention these aspects, indicating narrower generalizations than the ones we reported focusing on sample nationality. Future research that codes for the reporting of these other demographic dimensions is desirable.

Furthermore, unlike previous studies which also extracted overall counts of result claims to compare them with the counts of problematic generalizations [15], we only extracted and counted generalized claims. This limits our analyses, as we cannot present the proportionalities of restricted versus generalized claims.

Additionally, we focused on only four of the highest impact medical journals, our results may not generalize to the medical literature at large. However, assuming that top journals have as strict editorial policies as lower-ranking journals, our estimates are likely conservative.

Also, our use of country or region as proxies for national diversity ignores expatriates or mixed national demographics and other diversity dimensions, limiting generalizability. Relatedly, some Western countries may be highly diverse such that broad generalizations from Western samples may not always be problematic. However, since many clinical differences between Western and non-Western populations have been reported, invariance in relevant features across these populations cannot be assumed but should be examined [3,40–42].

While we recommend more diverse sampling, limited research resources may often be an obstacle. To counteract overly broad generalizations and generics use in medical research, we therefore suggest that when medical researchers extrapolate results, they follow the AMA guideline and specify to which study population(s) their findings most directly pertain (e.g., “US frontline workers with SARS-CoV-2 infections”) or use past tense or explicitly quantified claims about their study or target population instead of generic generalizations [15,17,38].

## Conclusions

To our knowledge, this is the first systematic analysis of the extent to which the medical literature contains hasty generalizations. About half of prospective studies published between 2022 and 2023 in the four highest impact medical journals contained generalizations beyond their study populations, but there was no evidence that articles with such generalizations had larger, more nationally diverse samples. Since most articles also did not report external validity strengthening factors to warrant such extrapolations, our results highlight significant methodological shortcomings in many available clinical studies. Finally, across four top medical journals, articles frequently contained conclusions with generics, which gloss over variations between people and can cause misunderstanding about treatments. It may therefore be advisable for medical researchers, reviewers, and editors to scrutinize more closely the generalizations in clinical studies, as overly broad extrapolations of results can adversely impact the translation of research into practice.

## Supporting information

S1 Checklist PRISMA 2020 checklist. (DOCX)

S1 Table Descriptive data for each of the four trial types. Studies without details on trial phase (*n* = 199) are omitted.
(DOCX)

S2 Table Number of countries and regions in the articles. (DOCX)

S3 Table Distribution of sample diversity in the restricted and generalized articles in numbers. (DOCX)

S4 Table Number of articles per medical sub-discipline (all journals). (DOCX)

S5 Table Results of Mann-Whitney *U* tests comparing the impact (RCR) of articles by journal. (DOCX)

S1 Fig World map illustrating the Western/non-Western origin of the samples from all the reviewed studies and the frequency with which the samples were recruited. Darker color indicates higher frequency of recruitment. Figure is self-created using Excel.
(DOCX)

S2 Fig Interval plot showing the mean overall relative citation rate (RCR) of each journal’s articles. The RCR is the observed citation count (raw citation count) divided by expected citation rate (expected citation count in the year the paper was published).
(DOCX)
