# Supplementary material for: Hasty generalizations and generics in medical research: A systematic review
Source: PLoS One. 2024 Jul 5;19(7):e0306749. doi: 10.1371/journal.pone.0306749 (PMC11226088; doi:10.1371/journal.pone.0306749)
Supplement: S1 Table — Studies without details on trial phase (n = 199) are omitted. (DOCX) [file pone.0306749.s002.docx]

**S1 Table.** Descriptive data for each of the four trial types. Studies without details on trial phase (*n* = 199) are omitted.

| **Trial type (N)** | **Phase I (9)** | **Phase II (64)** | **Phase III (235)** | **Phase IV (26)** |
| --- | --- | --- | --- | --- |
| Median # of participants (IQR) | 60.0 (20.5–169.5) | 150.0 (62.0–328.75) | 787.0 (357.0–1658.0) | 1203.0 (690.25–4371.50) |
| Median # of countries/regions (IQR) | 1.0 (1.0–1.0) | 3.0 (1.0–7.75) | 5.0 (1.0–15.0) | 1.0 (1.0–2.25) |
| Sample composition (N, %) |  |  |  |  |
| Non-Western | 0 | 6 (9.4) | 30 (12.8) | 7 (26.9) |
| Western | 9 (100) | 42 (65.6) | 94 (40.0) | 17 (65.4) |
| Mixed | 0 | 16 (25.0) | 111 (47.2) | 2 (7.7) |
| Generalized articles (N) (%) | 9 (100) | 36 (56.3) | 131(55.7) | 13 (50) |
| Unrestricted | 7 (77.8) | 25 (39.1) | 68 (28.9) | 6 (23.1) |
| Hedged | 4 (44.4) | 20 (31.3) | 56 (23.8) | 2 (7.7) |
| Practice-related | 1 (11.1) | 2 (3.1) | 22 (9.4) | 0 |
| Articles with generics | 9 (100) | 35 (54.7) | 98 (41.7) | 5 (19.2) |
| External validity | 5 (55.6) | 35 (14.9) | 131 (55.7) | 21 (80.8) |
| Limitations reported |  |  |  |  |
| Strengthening factors reported | 1 (11.1) | 5 (2.1) | 51 (21.7) | 6 (23.1) |
