# Supplementary material for: Hasty generalizations and generics in medical research: A systematic review
Source: PLoS One. 2024 Jul 5;19(7):e0306749. doi: 10.1371/journal.pone.0306749 (PMC11226088; doi:10.1371/journal.pone.0306749)
Supplement: S2 Table — (DOCX) [file pone.0306749.s003.docx]

**S2 Table.** Number of countries and regions in the articles.

| **Country** | **N** | **Country** | **N** | **Country** | **N** | **Country** | **N** |
| --- | --- | --- | --- | --- | --- | --- | --- |
| USA | 273 | India | 28 | Belarus | 5 | Albania | 1 |
| UK | 176 | Finland | 28 | Malawi | 5 | Honduras | 1 |
| Canada | 129 | Ukraine | 27 | Moldova | 5 | Oman | 1 |
| Spain | 120 | Turkey | 24 | Tanzania | 4 | Eswatini | 1 |
| Germany | 113 | Ireland | 23 | Nigeria | 4 | Tunisia | 1 |
| France | 110 | Romania | 22 | Lebanon | 4 | Mongolia | 1 |
| Italy | 104 | Colombia | 22 | Pakistan | 4 | Panama | 1 |
| Australia | 102 | Chile | 22 | Kenya | 4 | Luxembourg | 1 |
| Netherlands | 92 | Greece | 21 | Egypt | 3 | Ecuador | 1 |
| Poland | 86 | Serbia | 21 | Ethiopia | 3 | Cameroon | 1 |
| Belgium | 81 | Portugal | 20 | Iran | 3 | Kazakhstan | 1 |
| China | 80 | Singapore | 19 | Kuwait | 3 | Sudan | 1 |
| Japan | 70 | Norway | 19 | Mali | 3 | DR of Congo | 1 |
| South Korea | 64 | Lithuania | 16 | Botswana | 3 | Papua New Guinea | 1 |
| Brazil | 61 | Slovakia | 15 | Zimbabwe | 3 | Guinea | 1 |
| Czechia | 61 | Malaysia | 14 | Rwanda | 3 | Liberia | 1 |
| Russia | 54 | Latvia | 14 | Zambia | 3 | Sierra Leone | 1 |
| Hungary | 50 | Thailand | 12 | Ghana | 2 | Dominican Republic | 1 |
| Taiwan | 50 | Croatia | 11 | Bosnia/Herzegovina | 2 | Burkina Faso | 1 |
| Denmark | 50 | Estonia | 11 | Slovenia | 2 | Philippines | 1 |
| Mexico | 49 | Peru | 11 | Benin | 2 | Different country count | 107 |
| Austria | 48 | Philippines | 10 | Mozambique | 2 | *Region | N |
| Israel | 47 | Georgia | 9 | Bangladesh | 2 | *Africa | 1 |
| Argentina | 46 | Saudi Arabia | 9 | Uzbekistan | 2 | *Oceania | 1 |
| South Africa | 40 | Uganda | 7 | Paraguay | 2 | *Asian-pacific | 1 |
| New Zealand | 39 | Nepal | 6 | Macedonia | 2 | *West Africa | 1 |
| Sweden | 36 | Indonesia | 6 | Iceland | 2 | *North America | 2 |
| Switzerland | 35 | Vietnam | 5 | Gambia | 2 | *South America | 2 |
| Bulgaria | 30 | Guatemala | 5 | Columbia | 2 | *Europe | 2 |
