# Supplementary material for: Hasty generalizations and generics in medical research: A systematic review
Source: PLoS One. 2024 Jul 5;19(7):e0306749. doi: 10.1371/journal.pone.0306749 (PMC11226088; doi:10.1371/journal.pone.0306749)
Supplement: S3 Table — (DOCX) [file pone.0306749.s004.docx]

**S3 Table.** Distribution of sample diversity in the restricted and generalized articles in numbers.

| **Sample composition** | **Restricted articles** | **Generalized articles** | **Total** |
| --- | --- | --- | --- |
| Non-Western | 25 | 47 | 72 |
| Mixed | 62 | 84 | 146 |
| Western | 161 | 154 | 315 |
| *Total* | 248 | 285 | 533 |
