# Supplementary material for: Hasty generalizations and generics in medical research: A systematic review
Source: PLoS One. 2024 Jul 5;19(7):e0306749. doi: 10.1371/journal.pone.0306749 (PMC11226088; doi:10.1371/journal.pone.0306749)
Supplement: S4 Table — (DOCX) [file pone.0306749.s005.docx]

**S4 Table.** Number of articles per medical sub-discipline (all journals).

| **Medical sub-discipline** | **N** | **Medical sub-discipline** | **N** |
| --- | --- | --- | --- |
| Infectious disease | 95 | Nephrology | 11 |
| Oncology | 72 | Orthopedics | 11 |
| Cardiology and cardiac surgery | 59 | General surgery | 8 |
| Obstetrics and gynecology | 31 | Nutrition and metabolism | 8 |
| Neurology and neurosurgery | 29 | Allergy and immunology | 6 |
| Pediatrics | 27 | Ophthalmology | 6 |
| Respiratory medicine | 25 | Geriatrics | 5 |
| Endocrinology | 20 | Urology | 4 |
| Critical care/emergency medicine | 20 | Genetics | 4 |
| Dermatology | 17 | Hematology and oncology | 4 |
| Hematology | 15 | Anaesthesia and analgesia | 4 |
| Gastroenterology | 14 | Physical medicine and rehabilitation | 3 |
| Psychiatry | 12 | Vascular surgery | 3 |
| Rheumatology | 12 | Public health | 3 |
| Nephrology | 11 | Otolaryngology | 2 |
| Orthopedics | 11 | General medicine | 2 |
| General surgery | 8 | Radiology and imaging | 1 |
