# Supplementary material for: Hasty generalizations and generics in medical research: A systematic review
Source: PLoS One. 2024 Jul 5;19(7):e0306749. doi: 10.1371/journal.pone.0306749 (PMC11226088; doi:10.1371/journal.pone.0306749)
Supplement: S5 Table — (DOCX) [file pone.0306749.s006.docx]

**S5 Table.** Results of Mann-Whitney *U* tests comparing the impact (RCR) of articles by journal.

| **Journal comparison** | ***Mean ranks*** | ***U*** | ***z*** | ***p*** | ***r*** |
| --- | --- | --- | --- | --- | --- |
| *Lancet* vs. *NEJM* | 159.18 vs. 200.96 | 12434.00 | – 3.656 | < 0.001 | – 0.23 |
| *Lancet* vs. *JAMA* | 139.20 vs. 109.08 | 5894.00 | – 3.261 | 0.001 | 0.24 |
| *Lancet* vs. *BMJ* | 110.59 vs. 65.14 | 2032.50 | – 5.03 | < 0.001 | 0.47 |
| *NEJM* vs. *JAMA* | 193.24 vs. 120.30 | 7127.50 | – 6.43 | < 0.001 | 0.43 |
| *NEJM* vs. *BMJ* | 157.44 vs. 74.20 | 2522.00 | – 6.74 | < 0.001 | 0.59 |
| *JAMA* vs. *BMJ* | 89.45 vs. 68.35 | 2206.00 | – 2.674 | 0.007 | 0.26 |
