# Supplementary material for: Hasty generalizations and generics in medical research: A systematic review
Source: PLoS One. 2024 Jul 5;19(7):e0306749. doi: 10.1371/journal.pone.0306749 (PMC11226088; doi:10.1371/journal.pone.0306749)
Supplement: S1 Fig — Darker color indicates higher frequency of recruitment. Figure is self-created using Excel. (DOCX) [file pone.0306749.s007.docx]

**
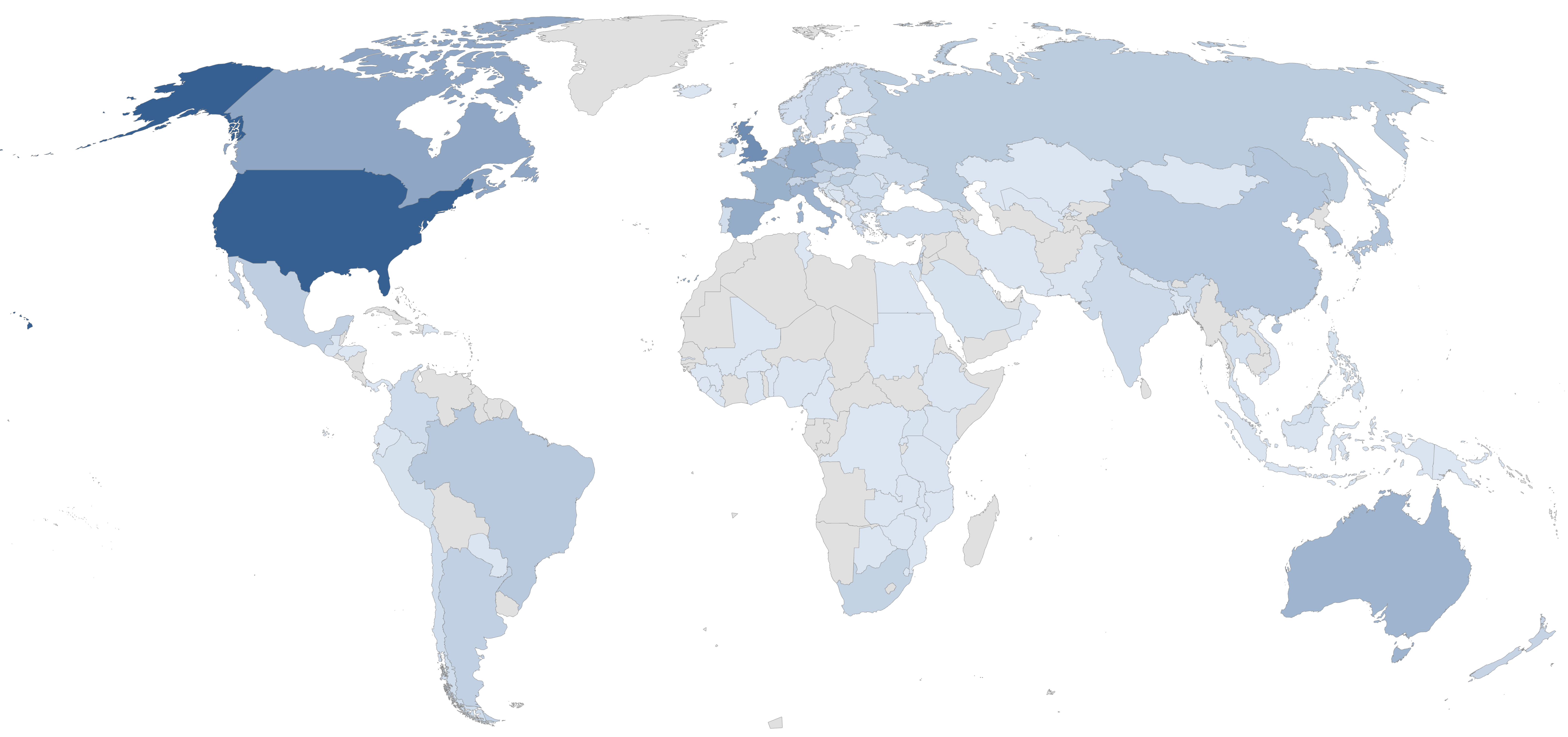
S1 Fig.** World map illustrating the Western/non-Western origin of the samples from all the reviewed studies and the frequency with which the samples were recruited. Darker color indicates higher frequency of recruitment. Figure is self-created using Excel.
