# Supplementary material for: Hasty generalizations and generics in medical research: A systematic review
Source: PLoS One. 2024 Jul 5;19(7):e0306749. doi: 10.1371/journal.pone.0306749 (PMC11226088; doi:10.1371/journal.pone.0306749)
Supplement: S2 Fig — The RCR is the observed citation count (raw citation count) divided by expected citation rate (expected citation count in the year the paper was published). (DOCX) [file pone.0306749.s008.docx]

**S2 Fig.** Interval plot showing the mean overall relative citation rate (RCR) of each journal’s articles. The RCR is the observed citation count (raw citation count) divided by expected citation rate (expected citation count in the year the paper was published).


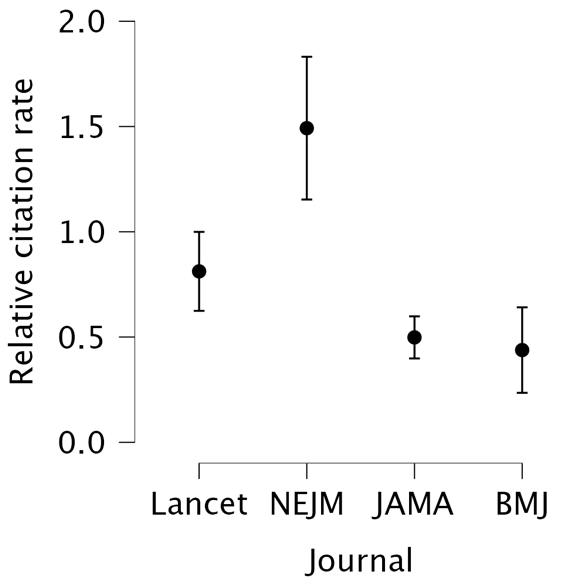


As indicated in Figure S2, articles from the four journals differed significantly in impact (normalized citation count), with *NEJM* articles having the highest impact (*Mean rank* = 322.64) followed by *Lancet* (*Mean rank* = 266.97), *JAMA* (*Mean rank* = 207.82), and *BMJ* (*Mean rank* = 152.69) (*H*(3) = 75.76, *p* < 0.001). S5 Table shows the results of the post-hoc comparisons.
